# Effects of size‐ and sex‐selective harvesting: An integral projection model approach

**DOI:** 10.1002/ece3.5719

**Published:** 2019-10-29

**Authors:** Marlene Wæge Stubberud, Yngvild Vindenes, Leif Asbjørn Vøllestad, Ian J. Winfield, Nils Christian Stenseth, Øystein Langangen

**Affiliations:** ^1^ Department of Biosciences Centre for Ecological and Evolutionary Synthesis (CEES) University of Oslo Oslo Norway; ^2^ Lake Ecosystems Group Centre for Ecology & Hydrology Lancaster Environment Centre Lancaster UK

**Keywords:** integral projection model, management strategies, mating function, pike, population dynamics, selective harvesting, sexual size dimorphism, two‐sex model

## Abstract

Harvesting is often size‐selective, and in species with sexual size dimorphism, it may also be sex‐selective. A powerful approach to investigate potential consequences of size‐ and/or sex‐selective harvesting is to simulate it in a demographic population model. We developed a population‐based integral projection model for a size‐ and sex‐structured species, the commonly exploited pike (*Esox lucius*). The model allows reproductive success to be proportional to body size and potentially limited by both sexes. We ran all harvest simulations with both lower size limits and slot limits, and to quantify the effects of selective harvesting, we calculated sex ratios and the long‐term population growth rate (*λ*). In addition, we quantified to what degree purely size‐selective harvesting was sex‐selective, and determined when *λ* shifted from being female to male limited under size‐ and sex‐selective harvesting. We found that purely size‐selective harvest can be sex‐selective, and that it depends on the harvest limits and the size distributions of the sexes. For the size‐ and sex‐selective harvest simulations, *λ* increased with harvest intensity up to a threshold as females limited reproduction. Beyond this threshold, males became the limiting sex, and *λ* decreased as more males were harvested. The peak in *λ*, and the corresponding sex ratio in harvest, varied with both the selectivity and the intensity of the harvest simulation. Our model represents a useful extension of size‐structured population models as it includes both sexes, relaxes the assumption of female dominance, and accounts for size‐dependent fecundity. The consequences of selective harvesting presented here are especially relevant for size‐ and sex‐structured exploited species, such as commercial fisheries. Thus, our model provides a useful contribution toward the development of more sustainable harvesting regimes.

## Graphical abstract

Harvesting often targets animals of certain sizes, and for many species, it also targets a certain sex. We quantify the effects of size‐ and sex‐selective harvesting by simulating different scenarios in an exploited pike population. Our new two‐sex model could aid in sustainable management of harvested species.

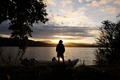

## 1 INTRODUCTION

In many exploited populations, harvesting is size‐selective (Fenberg & Roy, 2008; Zhou et al., 2010). Sexual size dimorphism is widespread in animals (Andersson, 1994; Fairbairn, Blanckenhorn, & Szekely, 2007), yet most of our knowledge on the effects of selective harvesting of sex‐structured species is derived from one‐sex or asexual demographic population models (but see Lindström, 1998). A common assumption in these models is *female dominance*; that is, population dynamicsare determined by females alone, and there are always enough males present for fertilization (Andersson, 1994; Caswell, 2001). For many species, this assumption may be too simplistic as males can impact female vital rates (e.g., Gerber & White, 2013; Magurran & Seghers, 1994; Mysterud, Coulson, & Stenseth, 2002; Rankin & Kokko, 2007; Reynolds, Mace, Redford, & Robinson, 2001; Wedell, Gage, & Parker, 2002) and vice versa. Two‐sex models may also provide more precise population projections as many species have sex‐specific vital rates (Fairbairn et al., 2007; Trivers, 1972). When individuals of a certain size and/or sex are harvested, it can result in detrimental changes in the population growth rate, structure, and sex ratio (e.g., Fenberg & Roy, 2008; Ginsberg & Milner‐Gulland, 1994; Greene, Umbanhowar, Mangel, & Caro, 1998; Hamilton et al., 2007; Kendall & Quinn, 2012; Milner, Nilsen, & Andreassen, 2007; Milner‐Gulland et al., 2003; Mysterud et al., 2002; Sato, Ashidate, Wada, & Goshima, 2005), which are not detectable in one‐sex or asexual models. Size‐structured dynamics are increasingly studied using integral projection models (IPMs; Easterling, Ellner, & Dixon, 2000; Ellner, Childs, & Rees, 2016). So far, few applications of this framework include both sexes and those that do assume female dominance (e.g., Plard, Schindler, Arlettaz, & Schaub, 2017; Schindler, Neuhaus, Gaillard, & Coulson, 2013). Here, we develop a size‐ and sex‐structured IPM that relaxes this assumption.

Body size tends to be positively correlated with reproductive success and survival in both sexes (Fairbairn et al., 2007). For many species, a female's fertility is generally limited by her fecundity, while a male's fertility is limited by his access to females rather than his gamete production (Trivers, 1972). The fertility of individuals of the same sex and individuals of the opposite sex can be affected if the survival of one sex decreases due to selective harvesting, or other factors such as disease (Guerra‐Silveira & Abad‐Franch, 2013), predation (Boukal, Berec, & Krivan, 2008), or environmental factors (Bodkin, Burdin, & Ryazanov, 2000). Males and females in harvested populations can be targeted differently due to sex‐specific size or shape (Fairbairn et al., 2007; Kendall & Quinn, 2012), behavior (e.g., sex‐specific depth preferences and arrival time at spawning ground; Fevolden, Westgaard, & Pedersen, 2015), or human preference (e.g., trophy hunting; Ginsberg & Milner‐Gulland, 1994; Milner‐Gulland et al., 2003). When one sex is harvested disproportionately, it can have detrimental consequences for the population (Fenberg & Roy, 2008). If the operational sex ratio (i.e., the ratio of males to females ready to mate; Emlen & Oring, 1977) is altered either way, it can affect both long‐term population growth rate (*λ*) and population structure.

For several harvested species, for example, of crustaceans, bovids, and cervids, there are size‐ and sex‐selective harvest regulations, and they are often male‐biased (Clark & Tait, 1982). In general, both empirical and theoretical studies have found that a male‐biased harvest reduces mean male age and size, and that the number of sexually active males in the population decreases (Carver, Wolcott, Wolcott, & Hines, 2005; Fenberg & Roy, 2008; Giordano & Lutscher, 2011; McLoughlin, Taylor, & Messier, 2005; Mysterud et al., 2002; Sørdalen et al., 2018). A shift toward younger, smaller, and fewer males can have a negative impact on female fertility and population growth through sperm limitation, reduced birthweights, and delayed parturition dates (e.g. Milner et al., 2007; Milner‐Gulland et al., 2003; Mysterud et al., 2002; Sato et al., 2005).

Although many species reproduce well with a strongly female‐biased sex ratio (Rankin & Kokko, 2007), even highly polygynous species have a minimum ratio of males needed to maintain reproduction (see, Reynolds et al., 2001, and references therein). Sperm are produced in immense numbers compared to eggs, but females can become sperm limited (Wedell et al., 2002). For instance, in saiga antelope (*Saiga tatarica tatarica*; Milner‐Gulland et al., 2003), and perch (*Perca fluviatilis*; Langangen et al., 2011) there is evidence of population collapse most likely caused by selective harvesting of males. These examples highlight the need for models to describe the dynamics of sex‐structured exploited populations. Two‐sex models can also aid to identify thresholds in sex‐specific harvesting ratios where the population is likely to decline. In general, two‐sex models describe the population dynamics better and they can be used to test the accuracy and validity of simpler asexual or one‐sex models (e.g., Eberhart‐Phillips et al., 2017; Gerber & White, 2013).

Experimental culling often conflicts with other interests such as conservation and is usually not an option to test consequences of harvest strategies (but see, Pardo, Rosas, Fuentes, Riveros, & Chaparro, 2015). Therefore, structured demographic models are widely used to represent populations (Caswell, 2001; Eberhart‐Phillips et al., 2017; Gerber & White, 2013; Jenouvrier, Caswell, Barbraud, & Weimerskirch, 2010; Shyu & Caswell, 2018). These models can be used to simulate harvesting regimes and provide insight into how population dynamics and parameters are likely to change (e.g., Kendall & Quinn, 2012; McLoughlin et al., 2005). Both structured population‐based models (e.g., matrix models; Caswell, 2001) and individual‐based models are commonly used to simulate population dynamics (for a comparison, see Sable & Rose, 2008). Model choice depends on the complexity of research question and study system, the available data, and computing capacity. Individual‐based models (also called agent‐based models) allow for highly complex models, as all individuals and their vital rates are modeled explicitly, but they require large amounts of detailed data and computation power. Conversely, structured population‐based models require less detailed data, compute faster, and have lower complexity as individuals are grouped together and assigned vital rates based on one or several traits, for example, sex, age, and/or size. IPMs, a type of structured population‐based model, are gaining traction, as they are the continuous‐state analogue to matrix models and can be parametrized with observational data (Easterling et al., 2000; Ellner et al., 2016).

To investigate how selective harvesting affects the population dynamics of a size‐ and sex‐structured species, we used an IPM with size‐ and sex‐dependent vital rates. We developed a mating function where fertility increases with size and where both sexes can limit reproduction. This model is applicable to size‐structured species with annual reproduction. Here, we parametrize the model for pike (*Esox lucius*), partly with long‐term data from lake Windermere, United Kingdom. Pike is an increasingly popular model species in ecology and evolutionary studies (Forsman et al., 2015; Skov & Nilsson, 2018), as it is a widespread and relatively long‐lived fish, commonly exploited in fisheries. We apply this IPM to simulate size‐ and sex‐selective harvest scenarios of varying intensity and selectivity. Through these simulations, we want to determine and quantify how both unintentional and intentional sex‐selective harvesting can affect population structure and growth rate.

## 2 METHODS

Our modeling framework builds on the two‐sex IPM developed by Schindler et al. (2013), and the female‐based pike IPM developed by Vindenes et al. (2014). The model is generally applicable to size‐structured species where both male fertility and female fertility increase with size, and here, we have adapted it to pike. Vital rates and variables used in the model are provided in Table 1, and values and estimated fixed effects are provided in Tables 2 and 3. All calculations and analyses were done in R version 3.5.1 (R Core Team, 2018).

**Table 1:** Variables and vital rates in the two‐sex integral projection model

Variables		Description
*x*	Size (cm)	Size at time *t*, from 1 to 125
*y*	Size (cm)	Size at time *t* + 1
*i* = *f*,*m*	Sex	*f* denotes females and *m* denotes males
*N_i_*(*x*,*t*)	Size distribution	Population size distribution for sex *i* (*i* = *f*,*m*), at time *t*
*S_i_*(*x*)	Survival function	Survival probability for an individual of sex *i* and size *x*
*G_i_*(*y*;*x*)	Next year's size distribution	Distribution of *y*, for sex *i* given current size *x*, is lognormal with mean *μ_G_*_,_ *_i_*(*x*) and variance $\sigma_{G,i}^{2}\left(x\right)$
*M_i_*(*x*,*t*)	Mating function	Expected number of offspring age 1 per individual of sex *i* and size *x*
*R_i_*(*y*)	Offspring size distribution	Distribution of offspring age 1 of size *y* and sex *i* is lognormal with constant mean *μ_R_*_,_ *_i_* and variance $\sigma_{R,i}^{2}$
*h_i_*(*y*)	Harvest survival probability	Probability that an individual of sex *i* and size *y* survives the harvest event

**Table 2:** Parameter values used in the two‐sex integral projection model on pike

Parameters	Description	Value
*t*	Time step (year)	1
*b*	Batch size (eggs)	300
*r*	Fertilization probability	0.90
*c_i_*	Harvest mortality probability	0–0.40
*q*	Sex ratio at age 1 (proportion females)	0.50
S_1_	Survival probability from fertilized egg to age 1	6.23 × 10^−4^

**Table 3:** Estimated fixed effects of the linear mixed‐effects models used to estimate survival, growth, offspring size distribution, and number of egg batches and milt ejaculations, for individuals of size *x* and sex *i* (male *m* or female *f*)

	Intercept	Size	Size^2^	Sex^a^	Size:Sex^a^
logit *S*(*x*)^b^	11.50	0.44	−3.93 × 10^−3^		
*μ_G_*_,_ *_i_*(*x*)	22.84	0.84	−7.26 × 10^−4^	−0.50	−0.07
*μ_R_*_,_ *_i_*	23.34			−0.63	
ln *e_f_*(*x*)	−8.09	3.30			
ln *e_m_*(*x*)	−10.70	4.28			

The constant *SD* of the offspring size distribution is $\sigma_{R}$ = 3.52.

a The effect for males.

b Values from Vindenes et al. (2014).

### 2.1 Two‐sex integral projection model

In a size‐structured IPM, the population dynamics is described by four size‐dependent vital rate functions: (a) annual survival probability, (b) next year's size distribution given current size (i.e., within‐individual changes in the focal trait over time), (c) production of offspring entering next year's population, and (d) next year's offspring size distribution (i.e., initial trait assignment to offspring). In a two‐sex IPM, one or all of the vital rates may differ between the sexes. Reproduction, and thereby population growth, relies on the mating system and life history of the modeled species (Caswell, 2001; Rankin & Kokko, 2007). In a general two‐sex model, the effect of males on female reproduction is captured by a function referred to as the *marriage function* in human demography, and the *birth* or *mating function* in other species (Bessa‐Gomes, Legendre, & Clobert, 2010; Caswell, 2001). The mating function gives the expected number of offspring per female and can be modified to a wide variety of mating systems (see Bessa‐Gomes et al., 2010; Caswell, 2001; Schindler et al., 2013). Below we define a mating function where the number of offspring depends on the total fertility of both males and females.

We denote this year's size by *x*, next year's size by *y*, and use subscript *i* = *f* for females, and *i* = *m* for males. The first two vital rate functions in our two‐sex IPM, (a) survival and (b) growth, are assumed independent of the current size distribution in the population. For an individual of size *x* and sex *i*, the natural survival probability from one time step to the next is given by *S_i_*(*x*). The growth function *G_i_*(*y*;*x*) describes the distribution of next year's size *y* given this year's size *x*, with mean *μ_G,i_*(*x*) and variance $\sigma_{G,i}^{2}\left(x\right)$. The population size and sex distribution at time *t* is denoted as *N_i_*(*x*,*t*). We use a prereproductive annual census so that offspring are counted as (nearly) 1 year old. The third vital rate function, (c) the number of offspring of sex *i* produced by a female of size *x*, depends on the current population's size and sex distribution, and is given by the mating function *M_i_*(*x*,*t*) (see section 2.1.1). The last vital rate function, (d) next year's offspring size distribution, is described by the function *R_i_*(*y*) and assumed independent from the parental size distributions. For offspring of sex *i*, this function describes the distribution of offspring size *y*, with a constant mean *μ_R,i_*, and variance $\sigma_{R,i}^{2}$.

The total population size distribution at time *t* is the sum of the female and male size distributions, *N*(*x*,*t*) = *N*_f_(*x*,*t*) + *N_m_*(*x*,*t*). The population size distribution at time *t* + 1 is determined by the vital rates described above:

(1) $$\begin{array}{c} N\left(y,t+1\right)=\int_{0}^{\infty }S_{f}\left(x\right)G_{f}\left(y;x\right)N_{f}\left(x,t\right)dx+\int_{0}^{\infty }S_{m}\left(x\right)G_{m}\left(y;x\right)N_{m}\left(x,t\right)dx\\ +\int_{0}^{\infty }M_{f}\left(x,t\right)R_{f}\left(y\right)N_{f}\left(x,t\right)dx+\int_{0}^{\infty }M_{m}\left(x,t\right)R_{m}\left(y\right)N_{f}\left(x,t\right)dx \end{array}$$

The first two integrals in Equation 1 represent survival and growth for females and males, respectively. The last two integrals represent female and male offspring produced in the current year that survive to age 1. In our harvest simulations, we used Equation 1 with added harvesting mortality (see section 2.2), to project the population growth over multiple time steps. We built the IPM projection kernel in Equation 1 over a size range from 1 to 125 cm, and discretized the size distribution using 300 size bins of 0.41 cm. To avoid unintentional eviction of large individuals, we used the solution proposed by Williams, Miller, and Ellner (2012) and expanded the upper limit of the size range (largest individual in our data: 110 cm).

#### 2.1.1 Mating function

The mating function is the main new development of our model, and it depends on the amount of gametes produced in the population. We consider a simplified promiscuous mating system; that is, both sexes can have multiple partners during the mating season, and we assume that all available gametes in the population are mixed. This mating function applies to batch spawning species like pike (Craig, 1996), gives the expected number of offspring age 1 produced by a female of size *x*, and is defined as follows:

(2) $$M_{f}\left(x,t\right)=rbqS_{1}\cdot e_{f}\left(x\right)\cdot \min\left(1,\frac{B_{m}\left(t\right)}{B_{f}\left(t\right)}\right),$$

where *r* is the fertilization probability of an egg, *b* is the egg number in each batch, *q* is the proportion of female offspring, and S_1_ is the survival probability from a fertilized egg to age 1, assumed independent of sex (see Table 2 for parameter values in our pike example). A female of size *x* is expected to produce a number of egg batches, *e_f_*(*x*). The minimum function in Equation 2 allows both sexes to limit reproduction as it depends on the ratio of the total male to female fecundity. *B_m_*(*t*) is the total number of milt ejaculations in the population, calculated by integrating the number of milt ejaculations *e_m_*(*x*) over the male size distribution, and *B_f_*(*t*) is the total number of egg batches in the population, calculated by integrating *e_f_*(*x*) over the female size distribution (see Appendix 1 for details). If *B_m_*(*t*) is larger than *B_f_*(*t*), females limit reproduction and the dynamics are the same as in a female‐based model (see Appendix 2). Conversely, if *B_m_*(*t*) is smaller than *B_f_*(*t*), males will limit reproduction. By replacing *q* in Equation 2 with (1 − q), we obtain the mating function describing the number of males produced by a female of size *x*: *M_m_*(*x*,*t*). When the sex ratio of offspring is equal, *M_f_*(*x*,*t*) = *M_m_*(*x*,*t*).

### 2.2 Harvest simulations

We investigated the effects of selective harvesting on population growth rate (*λ*), population structure, and sex ratio in harvest. We started with uniform size distributions for both sexes and ran the model with no harvest mortality until the population reached a stable size and sex structure. This stable population structure was then used as the initial population distribution for the different harvest scenarios. Harvesting can be simulated in a number of ways; here, we used a sequential model, as annual harvest was assumed to occur for a short time period toward the end of the growth season. After natural survival and growth, we multiplied the population distribution by a harvest survival probability *h_i_*(*y*) = 1 − *c_i_* that depends on sex *i*, size *y*, and the constant harvest mortality probability *c_i_* as described below. For each scenario, population size was calculated using numerical projections of the model described by Equation 1. The protection of either small (lower size limits), or small and large individuals (slot limits), is common practices in management of harvested populations (e.g. Hixon, Johnson, & Sogard, 2014). See Table 4 for harvest scenarios and size limits used here.

**Table 4:** Harvest scenarios and the different size limits

Harvest scenario	Name	Size limit^a^ (cm)
Lower size limit	L_30_	*x* > 30
	L_40_	x > 40
	L_50_	x > 50
Slot	S_30_	20 < x<40
	S_40_	30 < x<50
	S_50_	40 < x<60

a *x* is size of individuals.

In one set of simulations, we applied size‐selective harvesting only, that is, *h_f_*(*y*) = *h_m_*(*y*) and *c_f_* = *c_m_*. We considered three scenarios with lower size limits at 30, 40, and 50 cm. Below the size limit, all individuals survive harvest: *c* = 0 and *h*(*y*) = 1. Above the size limit, the individuals have a probability of being harvested: *c* > 0 and *h*(*y*) = 1 − c. For all size limits, we applied *c* in the range from 0 to 0.40 with 0.01 increments, encompassing realistic ranges for exploited fish populations. We also considered three slot limit scenarios at 20–40 cm, 30–50 cm, and 40–60 cm. Here, *h*(*y*) = 1 outside the slot limits, and *h*(*y*) = 1 − *c* within the slot limits. In a second set of simulations, we considered sex‐selective harvesting with the same size limits as before. Now, the harvest survival probability *h_i_*(*y*) = 1 − *c_i_* differed between the sexes; *h_f_*(*y*) = 1 − *c_f_* for females, and *h_m_*(*y*) = 1 − *c_m_* for males. We kept the sum of harvest mortality probabilities *c_f_* + *c_m_* constant, so that *c_m_* = 0.4 − *c_f_* for all simulations. In the simulations, *c_m_* increased with increments of 0.01 from *c_m_* = 0, that is, all female harvest, to *c_m_* = 0.4, that is, all male harvest.

For each simulation, we calculated the sex ratio as proportion of males in the harvest by dividing the total number of harvested males by the total number of harvested individuals. We also estimated *λ* as the total population size at time *T* divided by the population size at time *T* − 1. In all simulations, we used *T* = 40 (after testing other values), as it allowed the model population to stabilize, and we assume that any major shifts in population structure and dynamics would be detected within a 40‐year interval (see McLoughlin et al., 2005).

### 2.3 Parametrization of the model for pike

Our two‐sex model is an extension of the female‐based pike model developed by Vindenes et al. (2014), with size described as body length (fork length, cm). The size‐specific survival probability is the same as in Vindenes et al. (2014). In addition, we estimated growth rate and offspring size distribution with sex as a predictor in linear mixed‐effects models (Pinheiro & Bates, 2000) and developed a mating function that depends on both sexes. Following Vindenes et al. (2014), year was included as a random effect to account for any temporal variation, and temperature and year were included as fixed effects. As neither temperature nor temporal changes were the focus in the current study, they were averaged out. For all regressions with temperature, we used the mean annual water surface temperature, 10.55°C. For all regressions with year, we used the mean year in the given data set. The constant year and temperature effects are included in the estimates in Table 3, but see Table A1 for all estimated fixed effects and standard errors.

**Figure 1:**
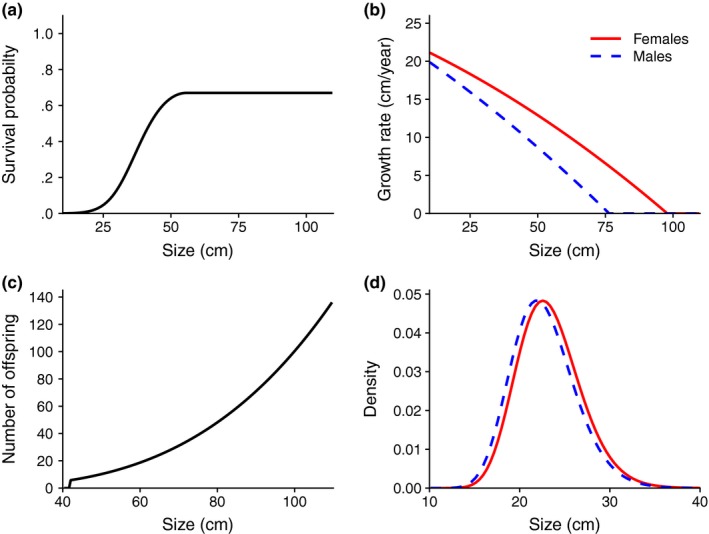
Vital rate functions for pike: (a) size‐dependent annual survival probability; (b) size‐dependent sex‐specific growth rate, where next year's size follows a truncated lognormal distribution with mean *μ_G_*_,_ *_i_*(*x*); (c) size‐dependent number of offspring age 1 for females when males do not limit reproduction; (d) sex‐specific offspring size distributions at age 1

The model was partly parametrized with data on pike from Windermere, where data have been collected since the 1940s (for more details, see Le Cren, 2001). We used four published data sets to estimate the different vital rates (URL in the references): (a) female fecundity (3,111 females, 1963–1996; Winfield, Fletcher, & James, 2013a), (b) male fecundity from weight data (4,168 males, 1963–1996; Winfield & James, 2018), (c) growth rate and offspring size distribution (7,939 females and 6,002 males, 1944–1995; Winfield, Fletcher, & James, 2013b), and (d) survival (3,992 individuals of both sexes, 1953–1990; Winfield, Fletcher, & James, 2013c). Fecundity and growth data were collected by gillnet sampling (Le Cren, 2001), while the survival data are from a capture‐mark‐recapture study (Kipling & Cren, 1984; Le Cren, 2001). All vital rate functions are described in Table 1 and shown in Figure 1 (with observed data in Figure A2).

The survival function *S*(*x*) is size‐dependent, assumed equal for both sexes, and set to be constant for large sizes after the maximum survival is reached (Figure 1a). As growth rates are sex‐specific, male and female pike will experience different survival probabilities at a given age. Next year's size distribution *y* given current size *x*, *G_i_*(*y*;*x*), is assumed to follow a truncated lognormal distribution with mean *μ_G,f_*(*x*) for females and *μ*_G,m_(*x*) for males (see Table 3). The variance in next year's size is given by the function $\sigma_{G}^{2}\left(x\right)=\tau \exp\left(2\delta x\right)$ (Pinheiro & Bates, 2000), where τ = 14.27 and δ = −0.01. Thus, the variance declines exponentially with size. The growth rates (cm/year) of both sexes decline with size and are non‐negative, and the difference between the sexes increases with size (Figure 1b), as females generally grow faster and to a larger size than males. Individuals can grow from size *x* to size *y* over one time step, but are not allowed to shrink (*y* ≥ *x*). The number of offspring age 1 produced by females increases with size (Figure 1c). The function is set to 0 below 42 cm as this is the average size at maturity for female pike in Windermere (Frost & Kipling, 1967), and we have no fecundity data for smaller females (Figure A2). The offspring size distribution *R_i_*(*y*) is assumed to be lognormal, independent of parent size, and with constant mean and variance for females (*μ_R_*_,_ *_f_*, $\sigma_{R}^{2}$) and males (*μ_R_*_,_ *_m_*, $\sigma_{R}^{2}$). Although the offspring size distributions (i.e., size at age 1) are similar for the sexes (Figure 1d), males have a slightly lower mean (Table 3 and Table A1).

As in many species with indeterminate growth, pike gonad size is positively correlated with body size (Craig, 1996). Thus, individual reproductive success is assumed to increase with size for both males and females. We estimated the expected gonad size given body size for both sexes, and as pike are batch spawners, we divided female gonads into egg batches, and male gonads into milt ejaculations. Both the number of egg batches *e_f_*(*x*) and milt ejaculations *e_m_*(*x*) were fitted as functions of size on log‐transformed data with linear models (See Table 3 and Appendix 1). For female pike, there are data on gonad size and egg numbers from Windermere (Winfield et al., 2013a). Given our data and the spawning behavior of pike, we assumed a batch size of 300 eggs (Clark, 1950; Fabricius & Gustafson, 1958; Frost & Kipling, 1967). There are no data on male gonads from Windermere, so we had to infer gonad and ejaculation size from data on male body weight (Winfield & James, 2018) and literature. Male pike gonad weight ranges from 2% to 4% of their total body weight, and we assumed a linear increase in gonads with body size and no sperm limitation in the study system. Together with results from a fertility study of a species with similar mating system (the common tench *Tinca tinca*; Targonska et al., 2016) and the optimal spermatozoa to egg ratio for fertilization (Lahnsteiner, Berger, & Weismann, 2003), we assumed an ejaculation size of 0.05 ml.

We assumed the batches and ejaculations were mated 1:1; that is, it takes one milt ejaculation to fertilize one egg batch. We excluded any spatial or temporal mating limitations by assuming that all mature individuals in the population arrive at the spawning ground at the appropriate time every year. By excluding mate finding and mate choice, any variation in reproductive success is determined by the size distributions of the two sexes. We could not estimate the first‐year survival S_1_ in Equation 2 due to lack of data. Instead, we adjusted the value of S_1_ to ensure that the predicted long‐term population growth rate of the simulated population without harvesting corresponded to the observed growth rate over the time series in the population (λ≈1.04; Langangen et al., 2011). With this approach, egg survival was set to 6.23 × 10^−4^ (Table 2), which is similar to values reported in other studies (Kipling & Frost, 1970; Vindenes et al., 2014).

## 3 RESULTS

**Figure 2:**
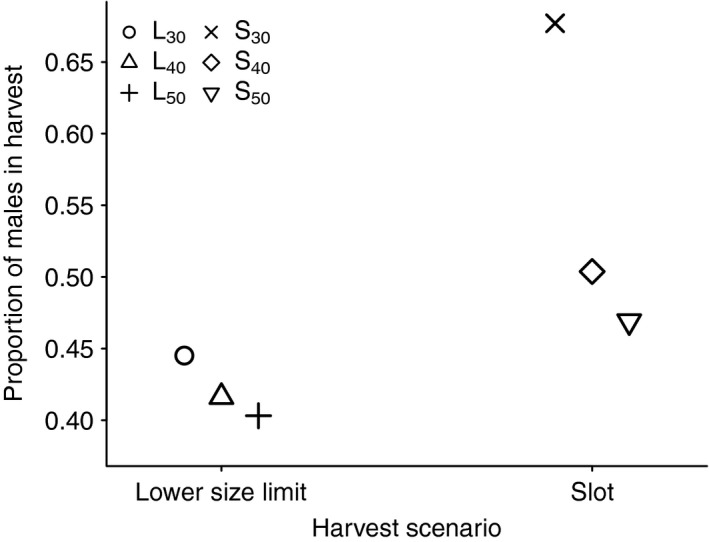
Proportion of males in harvest for size‐selective harvest simulations. Results for lower size limit are shown to the left and for slot limits to the right. There are three different size limits for each of the two harvest scenarios. See Table 4 for values of the different size limits

In the size‐selective harvest simulations with total annual harvest mortality probability up to 0.4, the sex ratio in harvest was independent of harvest mortality. The sex ratio in harvest depends on the size distributions of the two sexes, which is reduced but otherwise unchanged with size‐selective harvest intensity (Figure A3). The proportion of males in harvest was generally higher in the slot scenario and differed between the different size limits in both scenarios (Figure 2). In both scenarios, the male proportion was highest for the smallest size limits and it decreased with an increasing size limit to a greater degree in the slot than in the lower size limit scenario. For the size‐ and sex‐selective harvest simulations, the proportion of males in harvest increased with male harvest mortality probability for all size limits (Figure A4).

**Figure 3:**
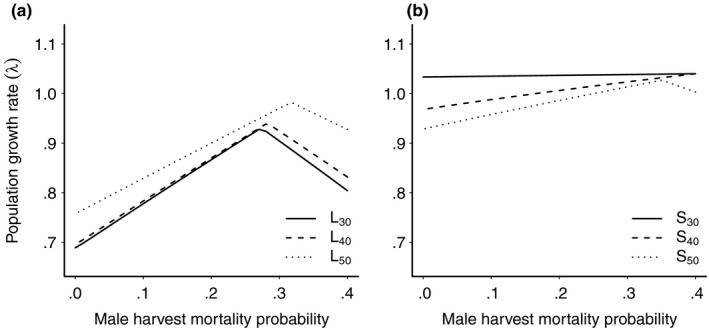
Long‐term population growth rate (*λ*) for size‐ and sex‐selective harvest simulations. Female and male harvest mortality probability vary inversely, but always sum up to 0.4. Lower size limit harvest scenario in panel (a), and slot scenario in panel (b). See Table 4 for values of the different size limits

For all size limits in the size‐selective harvest simulations, the population growth rate (*λ*) decreased with increasing total annual harvest mortality probability, and the decrease was steeper in the lower size limit than in the slot scenario (Figure A4). When the harvest simulations were both size‐ and sex‐selective, and the sum of female and male harvest mortality probabilities *c_f_* + *c_m_* was kept constant, *λ* increased as *c_m_* increased, and conversely *c_f_* decreased (Figure 3). In the lower size limit scenario, *λ* increased up to a maximum value for all three size limits. Beyond this point, *λ* decreased as *c_m_* continued to increase (Figure 3a). In the slot scenario, the population growth rate did not change as much with *c_m_* (Figure 3b), and only for the largest slot limit, S_50_, did we see a decrease in *λ* at high values of *c_m_*. The peak in *λ* signifies when the population growth shifts from being female to male limited. In the lower size limit scenarios, *λ* peaked at a male harvest proportion of 0.69 for L_30_, at 0.70 for L_40_, and 0.80 for L_50_. In the slot limit scenario, *λ* peaked at a male harvest proportion of 0.88 for S_50_.

## 4 DISCUSSION

By including both sexes and relaxing the assumption of female dominance, we have demonstrated how selective harvesting can affect size‐ and sex‐structured populations. Through harvest simulations of a sexually size dimorphic model species, we found that a purely size‐selective harvest regime can inadvertently be sex‐selective as well. For harvest simulations that were deliberately sex‐selective, a strongly male‐selective harvest shifted the long‐term population growth rate (*λ*) from being female to become male limited. Our results are based on a model parametrized for a relatively long‐lived gonochoristic (i.e., individuals do not change sex during their lifetime) batch spawning fish. Nevertheless, we argue that the general model structure is relevant to a range of size‐ and sex‐structured species. This includes many commercially exploited populations where both male fertility and female fertility increase with size.

The main new development of our model compared to earlier two‐sex IPMs is the mating function, which allows both males and females to limit reproduction. Reproductive output depends on the total fecundity of males relative to females, and the fecundities depend on the current size distribution of each sex. Individual reproductive success is therefore proportional to own size and relative to other individuals of both same and opposite sex. In our example, individual reproductive success corresponds to number of egg batches in females and number of milt ejaculations in males, both of which are determined by size. The mating function can be further modified to accommodate for how reproductive success could vary with size, for example, through mate choice or competition.

In the size‐selective harvest simulations, the lower size limit scenarios had a female‐skewed sex ratio in the harvest for all limits, while it was equal or male‐skewed in the slot limit simulations (Figure 2). Note that a comparison of the two harvest scenarios, lower size and slot limit, is limited. This is due to different size ranges and overall lower harvest pressure in the slot scenarios (see size distributions in Figure A3). These results demonstrate that purely size‐selective harvesting of a sexually size dimorphic species can be sex‐selective too. This was also found in a study of the moderately sexually size dimorphic Alaskan sockeye salmon (*Oncorhynchus nerka*), where size‐selective harvesting resulted in female‐biased adult sex ratios (Kendall & Quinn, 2012). Sex‐selective harvesting can be intentional through sex‐specific harvest limits or quotas (Clark & Tait, 1982; Sato et al., 2005), or unintentional due to sex‐specific behaviors and timing or location of harvest (Fevolden et al., 2015; Robinson et al., 2017).

For the size‐ and sex‐selective harvest simulations, *λ* increased with male harvest mortality probability and conversely reduced female harvest mortality probability, up to a threshold in the lower size limit scenario (Figure 3a). Beyond this threshold, *λ* declined as reproduction became sperm limited. The peak in *λ* increased with the size limit, as the male harvest proportion that maximizes *λ* depends on fertilization efficiency (Reynolds et al., 2001), which in our case is frequency dependent. In the slot scenario, *λ* peaked and decreased only for the largest size limit (Figure 3b). For all size limits where *λ* peaked, the maximum growth rate was reached at a high percentage of males in harvest, ranging from 70% to 90%.

Our harvest simulations indicate that a male‐biased harvest may be preferable in this pike population, at least up to a threshold. The threshold is important, as even in highly polygamous species there is a limit to the number of males required for reproduction (Rankin & Kokko, 2007; Reynolds et al., 2001). Intense male harvesting has led to severely reduced reproduction and population collapse in different species (e.g., Langangen et al., 2011; Milner‐Gulland et al., 2003; Reynolds et al., 2001). In perch, another temperate gonochoristic fish species, the population collapsed after a period of total annual harvest mortality at approximately 30%, where males constituted 80%–90% of the harvested individuals (Langangen et al., 2011). Sperm limitation may have played an important role in this collapse, and we note that both the total harvest mortality and the male proportion in the harvest in perch are comparable to the quantitative estimates from our harvest simulations where *λ* decreased.

**Figure A1:**
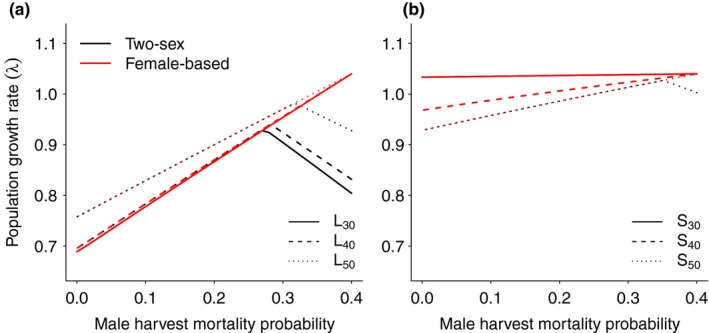
Long‐term population growth rate (*λ*) for size‐ and sex‐selective harvesting; a comparison of a female‐based and a two‐sex model. Harvest simulations with inversely varying female and male harvest mortality probability, and the total is constant at 0.4. Lower size limit harvest scenario are shown in panel (a), and slot scenario in panel (b). See Table 4 for values of the different size limits

**Figure A2:**
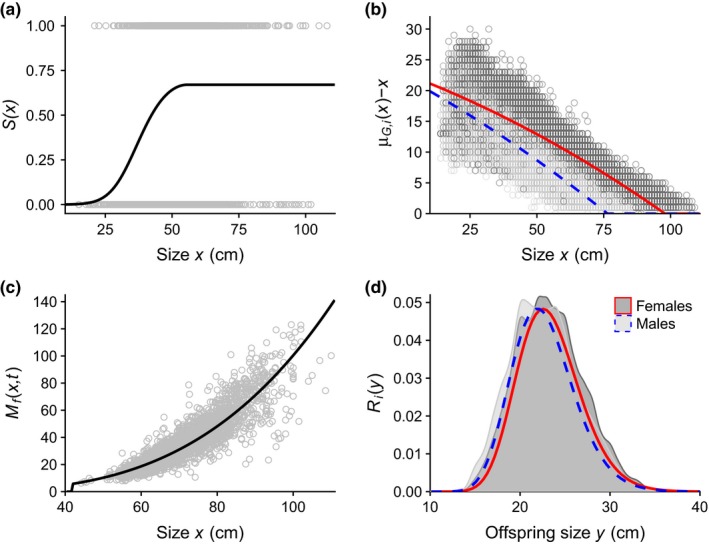
Vital rate functions for pike with data: (a) size‐dependent annual survival probability, *S*(*x*); (b) size‐dependent sex‐specific growth rate per year, where next year's size follows a truncated lognormal distribution with mean *μ_G_*,*_i_*(*x*), and *x* is current size; (c) size‐dependent number of offspring age 1 for females when males do not limit reproduction, *M_f_*(*x*,*t*); (d) sex‐specific offspring size distributions at age 1, *R_i_*(*y*)

**Figure A3:**
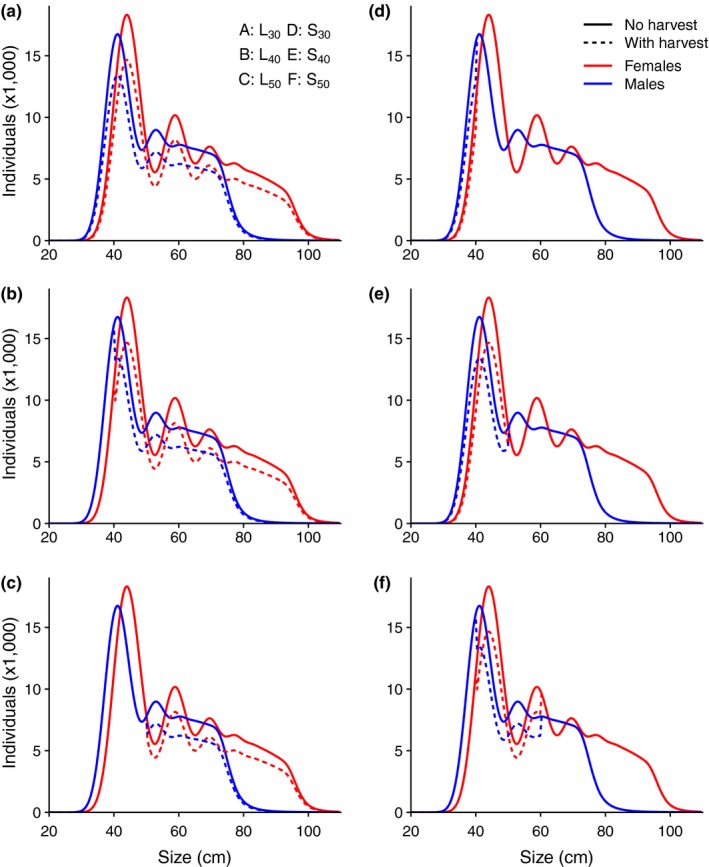
Size distributions for all the different size limits in the harvest simulations. The dashed lines are harvest simulations for equal harvest mortality for both sexes at 20%. Panels (a) to (c) are the different size limits in the lower size limit scenario, while panels (d) to (f) are the different size limits in the slot scenario. See Table 4 for values of the different size limits

**Figure A4:**
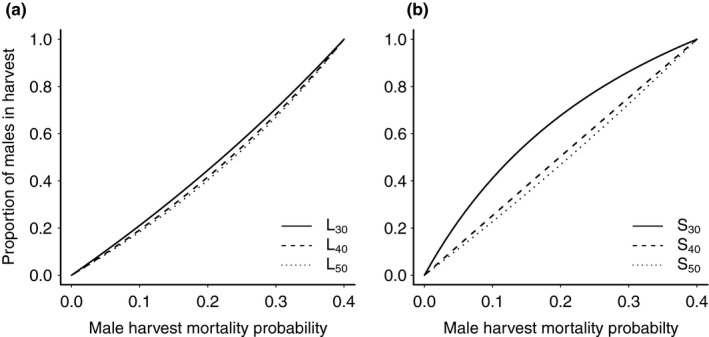
Proportion of males in harvest as a function of male harvest mortality probability. Size‐ and sex‐selective harvest simulations with inversely varying female and male harvest mortality probability, where the total is constant at 0.4. Lower size limit scenario in panel (a), and slot scenario in panel (b). See Table 4 for values of the different size limits

We compared the results from our two‐sex model with the result from a female‐based model (Appendix 2). As expected, the two models gave the same results for low‐to‐moderate male harvest mortality probabilities when female reproduction was unrestricted by males. In the female‐based model, *λ* continued to increase linearly for increasing male and conversely decreasing female harvest mortality probability, whereas it declined in the two‐sex model due to sperm limitation (Figure A1). This indicates that if males are targeted in harvested populations, they should be included in the population model as they might limit reproduction. The inclusion of both sexes has been shown to provide biologically more realistic population projections in different species (e.g., Eberhart‐Phillips et al., 2017; Gerber & White, 2013; Plard et al., 2017). Two‐sex models have also been suggested to be the preferred choice for both conservation (Reynolds et al., 2001) and biological control of populations (Rankin & Kokko, 2007).

There are two‐sex population models for harvested species that have indeterminate growth, where fecundity is positively correlated with body size, and that allow both sexes to restrict reproduction. But many of these models are individual‐based and require large amounts of detailed individual data and high computing capacity. Two‐sex individual‐based models have been used to study selective harvesting and sperm limitation in crustaceans (e.g., Rains, Wilberg, & Miller, 2018) and in sequential hermaphroditic vertebrates (i.e., species that change sex at a given size and/or sex ratio; Shapiro, 1987), mostly sex‐changing fish (e.g., Alonzo & Mangel, 2004; Robinson et al., 2017). Our model is adapted for gonochoristic species, as gonochorism is more common than hermaphroditism in not only temperate fish species (Warner, 1984), but also animals in general. For many study systems, data are collected on the population level, making population‐based models the appropriate choice. Individual‐ and population‐based models have been shown to be equally good at estimating population dynamics (Sable & Rose, 2008), but to our knowledge there are few, if any, population‐based two‐sex models for exploited gonochoristic vertebrates that can quantify how and when selective harvesting can lead to male limitation.

A general goal in management of exploited populations is to obtain large but sustainable catches over time (Reynolds et al., 2001). Here, we used *λ* as a crude measure to illustrate our approach. Managers of harvested populations often consider several other factors that affect long‐term sustainability, for example, environmental stochasticity. By using the same approach as we have outlined here, our model can also be used to simulate and optimize harvest strategies with respect to population parameters other than *λ*, for example, long‐term yield. An advantage of our two‐sex IPM is how easily the effects of different size‐ and/or sex‐selective harvesting scenarios can be evaluated.

Our size‐selective simulations show that certain size limits in harvesting regimes can be more or less sex‐specific (Figure 2). This is especially interesting for exploited populations where sex‐selective harvesting is unfeasible, for example, no sex‐specific temporal or spatial distribution, but the aim is to either harvest the sexes equally, or to harvest more or less of one sex. In accordance with other studies (e.g., Skonhoft, Yoccoz, Stenseth, Gaillard, & Loison, 2002), our results imply that if females limit male reproduction, more males than females should be harvested (assuming other potential effects of males can be ignored). In species where the opposite is true, it might be beneficial to selectively harvest females. Sex‐changing species represent a unique challenge for management (Alonzo & Mangel, 2004; Cote, 2003). They can be especially vulnerable to size‐selective harvesting (Hamilton et al., 2007), and the preferred sex‐selectivity in harvest would depend on the sex of the largest individuals. Many commercially exploited shrimp species in the Pandalidae family are protoandrous, that is, male to female (Charnov, 1982), while most hermaphroditic fish species are protogynous, that is, female to male (for and overview of hermaphroditic fish species, see De Mitcheson & Liu, 2008).

We applied lower size and slot limits in our selective harvest simulations, as these are common practices in management of size‐structured species. Intense harvesting of specific sizes, or a specific sex, can reduce reproductive output and shift size distributions and life‐history timing in the population (e.g., Ayllon et al., 2018; Carver et al., 2005; Hamilton et al., 2007; Mysterud et al., 2002; Sørdalen et al., 2018). These shifts could result in reduced population growth, whether they are genetic or phenotypically plastic (Fenberg & Roy, 2008). To mitigate such effects, a more holistic ecosystem approach has been advocated over the last decades (May, Beddington, Clark, Holt, & Laws, 1979). It argues for *balanced harvesting*, that is, nonselective harvesting across sizes, sex, species, and stocks (see Zhou et al., 2010). We did not look into balanced harvesting, but our model could be applied to test the effect of nonselective harvest too.

The results presented here, that is, the effects of long‐term selective harvesting, are obtained from a relatively simple frequency‐dependent model. Population growth rate is a reasonable measure of fitness for populations with weak density regulation, which seems to be the case for the adult pike population (>age 3) in Windermere. The pike population has fluctuated but steadily increased for decades, indicating that it has not yet reached a carrying capacity where density‐dependent factors would impair further population growth (Langangen et al., 2011; Vindenes, Langangen, Winfield, & Vøllestad, 2016). Density dependence is likely more important in the earlier life stages (Skov & Nilsson, 2018). In a potential future development, our two‐sex IPM can be expanded to incorporate density dependence in some or all vital rates. Then, a similar approach can be applied to test consequences of selective harvesting on population parameters such as carrying capacity. We assumed survival to be independent of sex, as there was no indication of sex‐specific differences in survival beyond that explained by size. We also followed Bessa‐Gomes et al. (2010), and only considered the effect of males on reproduction, ignoring other interactions between the sexes that could possibly affect population dynamics. Furthermore, we made some simplifying assumptions regarding the mating function. In particular, we assumed a promiscuous mating system without temporal or spatial restrictions, and equal mating probability for all mature individuals (a common assumption for species that aggregate to spawn, e.g., Alonzo & Mangel, 2004). As pike have homing behavior (Skov & Nilsson, 2018), and spawning is regulated by light and temperature (Craig, 1996), it is likely that our assumption of neither spatial nor temporal restrictions on mating is sound. There are several other factors that can limit reproduction besides time and space, for instance, endurance, gamete production, and behavior of both sexes (e.g., Milner‐Gulland et al., 2003; Mysterud et al., 2002), but for the aims of this study, it was reasonable to assume that all mature individuals were mated.

In future studies, it would be interesting to see how sex‐specific survival, other mating systems, and size‐ and/or sex‐dependent mating probabilities (see Bessa‐Gomes et al., 2010; Caswell, 2001; Cote, 2003; Schindler et al., 2013) would affect the population distributions and dynamics in a model without female dominance. Mating probability could also be adjusted to account for individual condition, size preferences, dominance, or other factors (see Plard et al., 2017; Schindler et al., 2013). To investigate potential eco‐evolutionary consequences, heritability and heterogeneity could be included in the model (Ayllon et al., 2018; Vindenes et al., 2016). Here, we focused on selective harvesting, but the presented model could easily be extended and applied to investigate the effects of any external driver on size‐ and sex‐structured populations.

We have shown how a two‐sex IPM can be used to simulate consequences of size‐ and/or sex‐specific harvesting. Such simulations can be used to improve management strategies and avoid population collapse or mitigate negative consequences of selective harvesting. The integral projection model framework is relatively simple, yet applicable to a wide range of species with size‐ and sex‐structured life histories. In conclusion, we believe that our model is a good starting point for further studies of two‐sex population dynamics in size‐structured populations, and that it could aid in developing better management strategies.

## CONFLICT OF INTEREST

None declared.

## AUTHORS' CONTRIBUTIONS

M.W.S led the writing of the manuscript, wrote scripts, and performed the analyses with guidance from Y.V. and Ø.L.; I.J.W. provided data collection, and together with L.A.V. contributed with advice and inputs on study system, species, and interpretation of results; Ø.L., Y.V., and N.C.S. conceived the research question and acquired funding. All authors contributed to the drafts and approved the final version for publication.

## Figures and Tables

**Figure A5:**
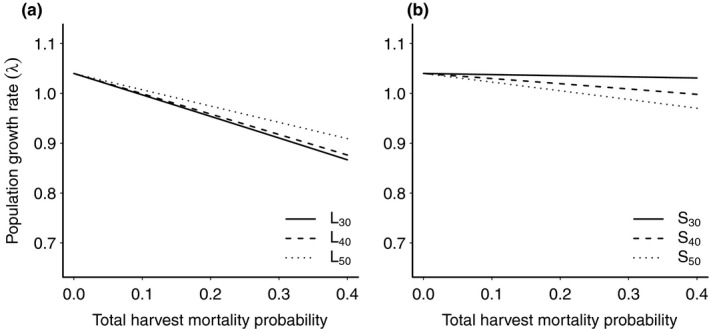
Long‐term population growth rate for size‐selective harvest simulations. Lower size limit harvest scenario in panel (a), and slot scenario in panel (b). See Table 4 for values of the different size limits

## Data Availability

Data available from the NERC Environmental Information Data Centre. Please see URLs in the references for the different data sets.

## APPENDIX 1

### EGG BATCHES AND MILT EJACULATIONS

For mature females, there are data on gonad weights (g) and estimated egg numbers for females of different sizes (Frost & Kipling, 1967; Winfield et al., 2013a). We know that pike only spawn during daytime (Clark, 1950), that they release on average two egg batches per minute (Fabricius & Gustafson, 1958), and that they spend approximately half the time resting. Based on our data and the spawning behavior of pike, we assumed an egg batch size of 300 eggs for all females over the whole spawning season. This allowed the largest females in our data to finish spawning within 10 days, which is the average time spent by females at the spawning ground (Clark, 1950; Frost & Kipling, 1967).

We inferred male gonad weight (g) from body weight (g), as male pike gonadosomatic index (GSI, gonad weight as percentage of total body weight; Craig, 1996) ranges from 2% to 4%, and it increases slightly for heavier males (Craig, 1996). We assumed that the GSI increased linearly with weight from 2% for the lightest mature male (340 g), to 4% for the heaviest (6,900 g) male in the data set (intercept: *β*_0_ = 0.02, weight: *β*_1_ = 3.05 × 10^−6^) Thus, we derived the estimated gonad weight given body weight (Winfield & James, 2018). Pike produce on average 0.95 ml milt per gram of gonad (see Craig, 1996 for details), but there are no data on ejaculation size. Based on a combination of results from a reproduction study on a species with smaller eggs but similar mating system to pike, the common tench (*Tinca tinca*, 0.05 ml milt was used to fertilize samples of 250—300 eggs; Targonska et al., 2016), and the optimal egg to spermatozoa ratio for fertilization (1:1.9 × 10^6^; Lahnsteiner et al., 2003), we assumed a milt ejaculation size of 0.05 ml. Number of ejaculations was then estimated by dividing the total amount of milt (ml) given gonad size by the assumed ejaculation size.

First, we log‐transformed the data and defined the expected number of batches and ejaculations produced by individuals of size *x*. We fitted linear models using generalized least squares (Pinheiro & Bates, 2000) with number of batches and ejaculations as functions of female and male size, respectively. The estimated fixed effects of the regression models (intercept: *β*_0_, size: *β*_1_) for egg batches and milt ejaculations are given in Table 3. The expected number of egg batches produced by a mature female (*x*_min_ = 42 cm) is given by

(A1) $$e_{f}\left(x\right)=\left\{\begin{array}{cc} \exp\left(\beta_{0}+\beta_{1}\ln x\right) & \text{if}\;x\geq x_{\min}\\ 0 & \text{otherwise}, \end{array}\right.$$

and the expected number of milt ejaculations produced by a mature male (*x*_min_ = 38 cm, i.e., the average size at maturity for males; Frost & Kipling, 1967) is given by

(A2) $$e_{m}\left(x\right)=\left\{\begin{array}{cc} \exp\left(\beta_{0}+\beta_{1}\ln x\right) & \text{if}\;x\geq x_{\min}\\ 0 & \text{otherwise} \end{array}\right.$$

The total number of available egg batches and milt ejaculations in the population depends on the size distributions of both sexes. The total number of egg batches available in the population is given by integrating Equation A1 over the female size distribution:

(A3) $$B_{f}\left(t\right)=\int_{0}^{\infty }N_{f}\left(x,t\right)\times e_{f}\left(x\right)dx,$$

and the total number of milt ejaculations available in the population is given by integrating Equation A2 over the male size distribution:

(A4) $$B_{m}\left(t\right)=\int_{0}^{\infty }N_{m}\left(x,t\right)\times e_{m}\left(x\right)dx$$

## APPENDIX 2

### FEMALE‐BASED MODEL

We ran size‐ and sex‐selective harvesting simulations on a female‐based model; that is, reproduction is not restricted by males. Then, the number of female offspring produced by a female of size *x* is given by

(A5) $$M_{f}\left(x,t\right)=rbqS_{1}\cdot e_{f}\left(x\right)$$

See Tables 2 and 3 for parameter values and descriptions. As expected, the two‐sex and the female‐based model gave the same results as long as females restrict reproduction; that is, males and sperm are abundant. Once there are too few males to fertilize all the egg batches, males restrict reproduction and the two‐sex model deviates from the female‐based one (Figure A1).

## APPENDIX 3

### ESTIMATED FIXED EFFECTS

Following the same method of model selection as in Vindenes et al. (2014), we estimated growth and offspring distribution with sex as an additional predictor. The estimated fixed effects of the regression models of the vital rate functions are given in Table A1. The survival function *S*(*x*) is assumed equal for both sexes (Table A1) and set to be constant for large sizes after the maximum survival is reached. For details on the estimation of survival, see Vindenes et al. (2014). Next year's distribution of size *y* given current size *x*, *G*_i_(*y*;*x*), is assumed to follow a truncated lognormal distribution with mean *μ_G_*_,_ *_f_*(*x*) for females, and *μ_G_*_,_ *_m_*(*x*) for males (see Table A1). Both means decline with size and are non‐negative, with a lower growth rate in males. The 95% confidence interval for the sex predictor (with female as the reference sex) does not include zero: 95% CI [−0.596, −0.412], indicating that growth rate is statistically different between sexes. The offspring size distribution at age 1 was assumed to be lognormal, with constant mean and variance: ($\mu_{R,f},\;\sigma_{R}^{2}$) and ($\mu_{R,m},\;\sigma_{R}^{2}$) for females and males, respectively (Table 3). The 95% confidence interval for the sex predictor (with female as the reference sex) does not include zero: 95% CI [−0.748, −0.511], indicating that offspring size distribution is statistically different between the sexes.

**Table A1:** Estimated fixed effects of the models used to estimate survival, growth, offspring size distribution, and number of egg batches and milt ejaculations

	Intercept	Sex	Size	Size^2^	Temp	Year	Size:Sex	Size:Temp	Size^2^:Temp
logit *S*(*x*)^a^	74.892 (3.569)		0.510 (0.038)	−0.004 (0.000)	0.193 (0.175)	−0.045 (0.001)		−0.007 (0.003)	
*μ_G_*_,_ *_i_*(*x*)	−53.178 (11.681)	−0.504 (0.047)	0.804 (0.023)	0.001 (0.000)	0.812 (0.151)	0.034 (0.006)	−0.075 (0.001)	0.003 (0.002)	−0.000 (0.000)
*μ_R_*_,_ *_i_*	−51.343 (20.626)	−0.629 (−0.061)			0.748 (0.256)	0.034 (0.011)			
ln *e_f_*(*x*)	−8.089 (0.117)		3.295 (0.028)						
ln *e_m_*(*x*)	−10.702 (0.092)		4.275 (0.022)						

Note

Each fixed effect is given with standard error in parentheses. Temp = temperature.

a Values from Vindenes et al. (2014).

## APPENDIX 4

### HARVEST SIMULATIONS

In the size‐selective harvest simulations, the proportion of males in harvest was independent of harvest intensity for all size limits. This can be explained by the size distributions (Figure A3). In the lower size limit scenario, all individuals above a certain size have a probability of being harvested, resulting in a reduction in number of individuals of both sexes at all larger sizes (Figure A3a–c). For the slot scenario, the size limit targeted only parts of the size distribution, and females and males were targeted differently given their sex‐specific growth rates and size distributions (Figure A3d–f).

In the size‐ and sex‐selective harvest simulations, the harvest mortality of both sexes varied inversely, but the total annual harvest mortality probability was always 40%. For all the size limits, the proportion of males in harvest increased with male harvest mortality probability (Figure A4). For the lower size limit scenario, there was little to no difference between the different size limits (Figure A4a). For the slot scenario, the proportion of males in harvest decreased slightly faster with increasing harvest mortality for the smallest size slot, S_30_ (Figure A4b).

For all size limits in the size‐selective harvest simulations, *λ* decreased with increasing total annual harvest mortality (Figure A5). In the lower size limit scenario, *λ* decreased faster for lower size limits, L_30_ and L_40_ (Figure A5a). For the slot scenario, the slopes were flatter, and *λ* decreased faster with increasing sizes, S_40_ and S_50_ (Figure A5b).
